# Relationship between social support, anxiety, and depression among frontline healthcare workers in China during COVID-19 pandemic

**DOI:** 10.3389/fpsyt.2022.947945

**Published:** 2022-09-14

**Authors:** Jie Zhan, Chen Chen, Xiaoting Yan, Xiaojing Wei, Lechang Zhan, Hongxia Chen, Liming Lu

**Affiliations:** ^1^Postdoctoral Research Station, Guangdong Provincial Hospital of Chinese Medicine, The Second Affiliated Hospital of Guangzhou University of Chinese Medicine, Guangzhou, China; ^2^Clinical Research and Data Center, South China Research Center for Acupuncture and Moxibustion, Medical College of Acu-Moxi and Rehabilitation, Guangzhou University of Chinese Medicine, Guangzhou, China; ^3^Department of Rehabilitation, Guangdong Provincial Hospital of Chinese Medicine, Guangzhou, China

**Keywords:** depression, anxiety, social support, COVID-19, frontline healthcare workers

## Abstract

**Background:**

Social support is an important factor affecting individual mental health. However, the relationship between social support and mental health in frontline healthcare workers (FHW) during the coronavirus disease 2019 (COVID-19) pandemic has garnered less attention. In this study, we aimed to investigate the level of social support and the prevalence of depression and anxiety in FHW during the COVID-19 pandemic and determine the factors affecting the relationship between social support, depression, and anxiety.

**Methods:**

A cross-sectional study using an online survey was conducted to collect data from FHW between 15 February and 31 March 2020 in China. The data included demographic factors, Self-rated Depression Scale (SDS), Self-rated Anxiety Scale (SAS), and Social Support Rate Scale (SSRS). Spearman correlation test was performed to determine the correlation among SAS, SDS, and SSRS scores. Multiple linear regression analysis was performed to determine the relationship among demographic factors, social support, depression, and anxiety in FHW.

**Results:**

Of all 201 participants, 44 (21.9%) had depressive symptoms and 32 (15.9%) had anxiety symptoms. The average total SSRS scores among FHW were lower than that of the norms of the Chinese general population (37.17 ± 7.54 versus 44.38 ± 8.38, *P* < 0.001). Marital status positively affected the SSRS score (β = 7.395, *P* < 0.01). Age over 40 years old negatively affected the SSRS score (β = −5.349, *P* = 0.017). The total SSRS score, subjective social support score, objective social support score, and support utilization score among FHW negatively correlated with the SAS score and SDS score (*P* < 0.05). A lower support utilization score was significantly associated with high anxiety and depressive symptoms (β = −0.869, *P* = 0.024; β = −1.088, *P* = 0.035, respectively).

**Conclusion:**

During the COVID-19 pandemic, FHW experienced depression, anxiety, and inadequate social support. The marital status and age had a major impact on social support. Social support was inversely associated with depression and anxiety. Improving the mental health of FHW by strengthening social support is crucial. Future studies are needed to investigate how to improve the level of social support and mental health condition of FHW facing public health emergencies in the future.

## Introduction

Since its outbreak in December 2019 in Wuhan, coronavirus disease 2019 (COVID-19) has posed a huge challenge to the healthcare system of China. On 29 January 2020, all 31 Chinese provinces declared public health emergencies and initiated lockdown policies in affected areas (1). As of 31 March 2020, 81,518 cases and 3,187 deaths were reported in China (2).

Amidst the development of the COVID-19 pandemic, frontline healthcare workers (FHW) globally were under tremendous pressure, and many suffered from psychological disorders (3), such as anxiety, depression, psychological stress response, and sleep disorders (4). When this online survey was conducted, the COVID-19 pandemic in China was still serious, with more than 2,400 confirmed cases and 139 deaths every day (5). FHW had to simultaneously prevent and treat the infection of COVID-19. The exhaustive work and risk of being infected by the virus caused a heavy psychological burden on FHW. Wearing protective equipment can relatively limit the communication of FHW. These factors can induce the presence of anxiety and depression among FHW during the COVID-19 pandemic. A cross-sectional study performed in the early stages of the COVID-19 outbreak reported that a significant proportion of FHW in China reported symptoms of depression (50.4%), anxiety (44.6%), insomnia (34.0%), and distress (71.5%) (6). Furthermore, anxiety symptoms can compromise work and frontline activities that can negatively affect private and social leisure activities (7). Previous studies focusing on the mental health reactions of healthcare workers during the acute phase of the COVID-19 pandemic reported that post-traumatic stress disorder, anxiety, and depression were associated with impairment in both work and social functioning (8, 9). FHWs are the direct providers of hospital services and are a key factor in controlling the pandemic (10). These psychological disorders affect the quality of life and health of FHW and also their professional performance, which greatly reduce their work efficiency and negatively affect the control of the COVID-19 pandemic.

Social support, which refers to the social connections, social integration, and major group relationships for individuals, is an important part of social psychology (11). Social support can enhance the protection of self-consciousness and effectively relieve the psychological disorders of individuals (12, 13). Lau proposed that social support is a crucial factor in alleviating stressful events and reducing their negative effects on the physical and mental health of individuals (14). Social support is associated with depression among health workers in developed countries (15, 16). Similarly, Chinese physicians had a higher prevalence of depressive symptoms and lower social support than the Chinese general population (17). A cross-sectional survey of Chinese doctors reported that social support is an important protective factor for the psychology of doctors. The more social support provided to doctors, the lower their depressive and anxiety symptoms (18). Another study also revealed that sufficient social support and training on positive coping skills can reduce anxiety in medical staff during the COVID-19 pandemic (19). Although these studies investigated the association between social support, depression, and anxiety among health workers, the relationship between social support and mental health among FHW during the COVID-19 pandemic was not investigated. The studies on psychological disorders of FHW in China mostly focused on epidemiological surveys (6, 20, 21). However, the relationship between social support and mental health among FHW during the COVID-19 pandemic has garnered less attention. The factors affecting the relationship between social support and mental health among FHW remains is yet to be investigated, which limits us from taking effective measures to help reduce psychological disorders, such as depression and anxiety, among FHW.

To bridge this gap, in this study, we aimed to investigate the level of social support and the prevalence of depression and anxiety among FHW during the COVID-19 pandemic and determine the factors affecting the relationship between social support, depression, and anxiety. We hope that our research can help us better understand the psychological needs of FHW during the pandemic and provide a basis for government health departments to formulate effective psychological rehabilitation intervention policies.

## Materials and methods

### Ethical approval

This study was conducted in accordance with the Declaration of Helsinki and approved by the Ethics Committee of Guangdong Provincial Hospital of Chinese Medicine (No. ZE2020-036). This trial has been registered at the Chinese Clinical Trial Registry (No. ChiCTR2000029815). All participants provided their informed consent prior to their participation in the electronic questionnaire (with a “yes or no” question) to confirm their willingness to participate in this study. The data was stored on a cloud server accessible only to the main author.

### Study design and participants

This cross-sectional study was conducted from 15 February to 31 March 2020 in China after the COVID-19 outbreak has been declared as a public health emergency of international concern. As the Chinese government advised the public to reduce their face-to-face interactions and tightened restrictions on the flow of people, potential participants were invited to complete an anonymous online questionnaire. The online questionnaire was developed using the SurveyStar^1^ (Changsha Ranxing Information Technology Co., Ltd., Changsha, China). Next, we shared the questionnaire on the social media, including WeChat and Tencent QQ. The responses to the questionnaire were automatically collected into an EXCEL spreadsheet by the SurveyStar for further data analyses.

The inclusion criteria for the participants were as follows: (1) being a FHW; (2) age >18 years; (3) Chinese resident; (4) no history of mental illness; (5) submitted only one survey using the same IP address; and (6) volunteered to participate in this study. The exclusion criteria included the following: (1) working time on the frontline of COVID-19 prevention and control <1 week; (2) trainee, interns, external hired, or dispatched personnel; (3) refusal to participate in the survey; and (4) the time to complete the questionnaire being <5 min.

To determine the practicability of the online questionnaire, the constituent instruments were pilot-tested beforehand on a group of 20 FHW, and these individuals were excluded from the main study.

### Survey development

#### Socio-demographic characteristics

Demographic and social data were self-reported by the participants, which included their age, gender, marital status (married or single), educational level (master’s degree or above, bachelor’s degree or lower), profession (doctor or nurse), seniority (primary, intermediate, or senior), and the number of days working at the frontline since COVID-19 outbreak (7–28, >28 days). The participants were asked whether they were currently working in one of the following three departments: fever clinics, isolation ward for suspected cases, and treatment ward for confirmed cases. The respondents who answered with a “yes” were defined as FHW and those who answered with a “no” were defined as second-line healthcare workers. The latter were excluded from this study.

### Depressive symptoms

Depressive symptoms of FHW were assessed by the well-established Self-rated Depression Scale (SDS) (22). SDS is a widely recognized tool for evaluating depressive disorder (23). The score of each item was added and then multiplied by 1.25 as a total score ranged from 25 to 100, with a higher score indicating more depressive symptoms. The severity rating index for SDS were as follows: normal (25–52), mild (53–62), moderate (63–72), and severe (73–100). We categorized FHW with an SDS score ≥53 as having depressive symptoms. In this study, the Cronbach’s alpha of SDS was 0.886.

#### Anxiety symptoms

Anxiety symptoms were assessed by using the Self-rated Anxiety Scale (SAS) (24, 25). An aggregate score of 20 items was multiplied by 1.25, with a higher score indicating more severe levels of anxiety. Anxiety score of <50 was considered normal, that of 50–60 was considered mild, that of 61–70 was considered moderate, and that >70 was considered severe. We set the cutoff point of SAS at 50 to suggest anxiety symptoms. In this survey, the Cronbach’s alpha of SAS was 0.806.

### Social support

Social support was assessed by using the Social Support Rate Scale (SSRS), which was designed to determine how much support respondents received from their family, friends, and social contexts (26, 27). SSRS consists of three subscales: subjective support (four items on the number of friends who offered assistance, relationship with neighbors, relationship with colleagues, and the level of support from family members), objective support (three items on the living conditions in the past year, problem-solving channels in emergency situations, and the sources of psychological comfort in the event of stress or resistance), and support utilization (three items on the way one expresses when in trouble, the way in which one seeks help when in trouble, and the willingness of participation in group activities). The total SSRS score is the sum of these three subscales scores, with a higher score indicating higher levels of social support (28). In this study, the Cronbach’s alpha value of SSRS was 0.844.

### Statistical analyses

The dataset was entered and analyzed using the statistical package for the social sciences (SPSS) version 25.0 (IBM Corp., Armonk, NY, United States). The following descriptive statistics were used, including frequencies (*n*), percentages (%), means, and standard deviations (SD).

Shapiro–Wilk test was performed to examine the normality. Reliability was assessed with Cronbach’s alpha. One-sample mean test was performed to identify the difference in the SSRS score between FHW and the norms of Chinese general population. One-way analysis of variance was conducted to identify the difference in the SDS, SAS, and SSRS of the FHW based on the demographic factors. Spearman correlation analyses were performed to examine the relationship among the scores of SAS, SDS, and SSRS.

Multiple linear regression analysis was performed to examine the association between social support and demographic factors. The dependent variable was the total SSRS score, and the independent variables included age, marital status, and seniority. We also conducted multiple linear regression analysis to identify the relation among social support and the SDS and SAS scores. The dependent variable was the SDS and SAS scores. The independent variables included subjective social support score, objective social support score, support utilization score, and the total SSRS score. The regression model was statistically significant (*P* < 0.05), indicating a linear correlation between the dependent and independent variables. All tolerance values were >0.1, and the VIF value was <10, which indicated that no data had multicollinearity (29). Regression coefficient estimates (β), standard error (SE) of β, 95% confidence intervals (CIs) of β, and *P*-values were also analyzed. *P* < 0.05 was considered to indicate statistical significance.

## Results

### Sample characteristics

A total of 211 participants answered questionnaires in the survey, of which 201 fulfilled the study inclusion criteria, giving an effective response rate of 95.3%. Of the 201 participants (mean age = 33.31 years, SD = 7.12 years), 100% worked on the frontline during the COVID-19 pandemic, 43.3% were between the ages of 30 and 39, 74.6% were women, 58.2% were married, and 82.1% had a bachelor’s degree or lower. In terms of professionally, 63.2% were registered nurses, 55.2% held a primary professional title, 47.8% of healthcare workers were affiliated with the suspected case isolation ward unit, and 73.6% worked on the frontline for 7–28 days. The socio-demographic and clinical characteristics of the participants are presented in Table 1.

**TABLE 1:** The socio-demographic and clinical characteristics of the participants.

Characteristic	Total sample	Depressive symptoms	Anxiety symptoms
Participants	201 (100%)	44 (21.9%)	32 (15.9%)
**Age, *n* (%)**			
20–29	74 (36.8%)	13 (29.55%)	10 (31.25%)
30–39	87 (43.3%)	21 (47.73%)	12 (37.50%)
>40	40 (19.9%)	10 (22.72%)	10 (31.25%)
**Gender, *n* (%)**			
Male	51 (25.4%)	11 (25.00%)	10 (31.25%)
Female	150 (74.6%)	33 (75.00%)	22 (68.75%)
**Marital status, *n* (%)**			
Single	84 (41.8%)	16 (36.36%)	12 (37.50%)
Married	117 (58.2%)	28 (63.64%)	20 (62.50%)
**Education, *n* (%)**			
Bachelor’s degree or lower	165 (82.1%)	39 (88.64%)	27 (84.38%)
Master’s degree or above	36 (17.9%)	5 (11.36%)	5 (15.62%)
**Profession, *n* (%)**			
Doctor	74 (36.8%)	12 (27.27%)	11(34.37%)
Nurse	127 (63.2%)	32 (72.73%)	21(65.63%)
**Seniority, *n* (%)**			
Primary	111 (55.2%)	25 (56.82%)	14 (43.75%)
Intermediate	58 (28.9%)	13 (29.55%)	13 (40.63%)
Senior	32 (15.9%)	6 (13.63%)	5 (15.62%)
**Department, *n* (%)**			
Fever clinics	83 (41.2%)	21 (47.73%)	14 (43.75%)
Isolation ward for suspected cases	96 (47.8%)	18 (40.91%)	15 (46.87%)
Treatment ward for confirmed cases	22 (11.0%)	5 (11.36%)	3 (9.38%)
**Number of days on the frontline since the COVID-19 outbreak, *n* (%)**			
7–28 days	148 (73.6%)	27 (61.36%)	24 (75.00%)
>28 days	53 (26.4%)	17 (38.64%)	8 (25.00%)
SDS score, mean ± SD	43.30 ± 11.38	60.02 ± 6.19	
SAS score, mean ± SD	40.98 ± 8.20		54.47 ± 5.79

SAS, Self-rated Anxiety Scale; SDS, Self-rated Depression Scale; SD, standard deviation.

### Assessment of depression and anxiety

Among 201 FHW, the mean scores of SAS and SDS were 40.98 (SD = 8.20) and 43.30 (SD = 11.38), respectively. A total of 44 (21.9%) participants self-reported depressive symptoms, and 32 (15.9%) participants self-reported anxiety symptoms (Table 1).

**FIGURE 1:**
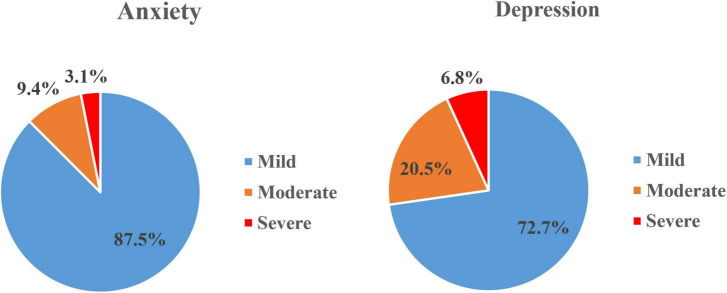
The distribution of levels of severity on depression and anxiety (prevalence of depressive and anxiety symptoms based on a cutoff score of 53 on SDS and 50 on SAS, respectively. Of 201 participants, 44 participants self-reported with mild-severe depressive symptoms and 32 participants self-reported with mild-severe anxiety symptoms).

In terms of depression and anxiety, the participants were distributed across the three levels of severity. Based on the data of this survey, 44 participants reported depressive symptoms, 32 (72.7%) reported mild depression, 9 (20.5%) reported moderate depression, and 3 (6.8%) reported severe depression. Of 32 participants with anxiety symptoms, 29 (90.6%) reported mild anxiety, 2 (6.3%) reported moderate anxiety, and 1 (3.1%) reported severe anxiety (Figure 1).

### Assessment of social support

The average scores of SSRS, subjective support, objective support, and support utilization were 37.17 (SD = 7.54), 20.20 (SD = 3.97), 9.41 (SD = 3.47), and 7.56 (SD = 2.02), respectively. Shapiro–Wilk test showed that the total SSRS score and three subscales scores of the SSRS were normally distributed (*P* > 0.05). The one-sample mean test showed that the total SSRS score and three subscales scores of the SSRS among FHW were lower than that of the norms of the Chinese general population (30), respectively (*P* < 0.01) (Table 2).

**TABLE 2:** The difference of social support scores among frontline healthcare workers and the norms of Chinese general population.

Variable	Total sample (*n* = 201)	Norms of general population	*t*	*P*-value
Total SSRS score, mean ± SD	37.17 ± 7.54	44.38 ± 8.38	−13.560	<0.001
Subjective social support score, mean ± SD	20.20 ± 3.97	23.81 ± 4.75	−12.896	<0.001
Objective social support score, mean ± SD	9.41 ± 3.47	12.68 ± 3.47	−13.341	<0.001
Support utilization score, mean ± SD	7.56 ± 2.02	9.38 ± 3.40	−12.781	<0.001

SD, standard deviation; SSRS, Social Support Rate Scale.

### The association between demographic factors and social support, depression, and anxiety

One-way analysis of variance showed no significant difference in the effect of demographic factors on depressive and anxiety symptoms (Table 3). However, a one-way analysis of variance showed that FHW within the age of 30–39 years old had a higher SSRS score compared with the younger FHW (39.22 ± 7.45 versus 34.62 ± 6.93, *P* < 0.01). Moreover, the total SSRS score of married FHW was higher than that of unmarried FHW (39.92 ± 6.73 versus 33.33 ± 6.93, *P* < 0.01). Compared with FHW with primary titles, FHW with senior titles had a lower total SSRS score (39.34 ± 7.34 versus 35.64 ± 7.72, *P* = 0.013), whereas FHW with intermediate titles had a higher total SSRS score than those with senior titles (38.90 ± 6.70 versus 35.64 ± 7.72, *P* = 0.007) (Table 3).

**TABLE 3:** Difference in total SSRS, SDS, and SAS based on demographic characteristics.

Variable	Total SSRS score (mean ± SD)	*F*	*P*-value	SAS score (mean ± SD)	*F*	*P*-value	SDS score (mean ± SD)	*F*	*P*-value
Total sample (*n* = 201)	37.17 ± 7.54			40.98 ± 8.20			43.30 ± 11.38		
**Age**									
20–29 (*n* = 74)	34.62 ± 6.93	7.982	<0.001	40.70 ± 7.45	0.780	0.460	42.62 ± 10.49	0.651	0.523
30–39 (*n* = 87)	39.22 ± 7.45^a^			40.55 ± 8.52			43.05 ± 11.76		
>40 (*n* = 40)	37.43 ± 7.59			42.43 ± 8.87			45.10 ± 12.22		
**Gender**									
Male (*n* = 51)	38.75 ± 7.52	3.016	0.084	41.92 ± 9.56	0.899	0.344	43.67 ± 11.25	0.071	0.790
Female (*n* = 150)	36.63 ± 7.50			40.66 ± 7.70			43.17 ± 11.46		
**Marital status**									
Single (*n* = 84)	33.33 **±** 6.93	45.704	<0.001	40.68 ± 8.70	0.194	0.660	43.11 ± 10.90	0.041	0.841
Married (*n* = 117)	39.92 **±** 6.73			41.20 ± 7.86			43.44 ± 11.76		
**Education**									
Bachelor’s degree or lower (*n* = 36)	37.14 **±** 7.51	0.001	0.979	39.11 ± 10.33	2.291	0.132	41.72 ± 11.30	0.840	0.360
Master’s degree or above (*n* = 165)	37.18 **±** 7.57			41.39 ± 7.64			43.64 ± 11.40		
**Profession**									
Doctor (*n* = 74)	38.03 **±** 7.24	1.520	0.219	39.82 ± 9.04	2.340	0.128	41.72 ± 10.80	2.278	0.133
Nurse (*n* = 127)	36.67 **±** 7.70			41.65 ± 7.63			44.22 ± 11.65		
**Seniority**									
Primary (*n* = 32)	39.34 ± 7.34^b^	5.362	0.005	39.72 ± 8.38	0.448	0.639	42.09 ± 12.23	0.470	0.626
Intermediate (*n* = 58)	38.90 ± 6.70^b^			41.26 ± 8.66			42.66 ± 10.80		
Senior (*n* = 111)	35.64 ± 7.72			41.20 ± 7.95			43.98 ± 11.48		
**Department**									
Fever clinics (*n* = 83)	37.67 **±** 8.34	1.277	0.281	41.07 ± 8.33	1.053	0.351	43.66 ± 12.00	0.194	0.824
Isolation ward for suspected cases (*n* = 96)	36.35 **±** 7.13			41.44 ± 8.33			43.29 ± 11.40		
Treatment ward for confirmed cases (*n* = 22)	38.82 **±** 5.75			38.64 ± 7.04			41.95 ± 9.03		
**Number of days on the frontline since the COVID-19 outbreak**									
7–28 days (*n* = 148)	37.20 ± 7.47	0.011	0.916	40.90 ± 8.70	0.055	0.815	42.89 ± 11.33	0.739	0.391
>28 days (*n* = 53)	37.08 ± 7.80			41.21 ± 6.69			44.45 ± 11.56		

One-way analysis of variance showed age, marital status, seniority had an effect on total SSRS score. SAS, Self-rated Anxiety Scale; SDS, Self-rated Depression Scale; SD, standard deviation; SSRS, Social Support Rate Scale. ^a^Compared with participants with 20–29 years old, P < 0.01. ^b^Compared with participants with senior title, P < 0.05.

Multiple linear regression analysis was performed to investigate the association between social support and demographic factors. The regression model was statistically significant [*F*(5,195) = 11.216, *P* < 0.001], which suggested that a linear correlation existed between the dependent and independent variables. In this study, all tolerance values were greater than 0.1 (minimum 0.3) and the VIF was less than 10 (maximum 3.5), which indicated that all data had no multicollinearity. Multiple linear regression analysis showed that being married positively affected the SSRS score (β = 7.395, *P* < 0.01), and age over 40 years old negatively affected the SSRS score (β = −5.349, *P* = 0.017). Multiple linear regression analysis also showed that age, marital status, and seniority were associated with social support, which explained 20.3% of all variance (Table 4).

**TABLE 4:** Regression analysis of the effects of demographic factors on social support.

Variable	Unstandardized coefficients		Standardized coefficients	*t*	*P*-value	95% confidence interval for β	
	β	SE				Lower bound	Upper bound
Constant	34.129	1.670		20.432	<0.001	30.834	37.423
**Age (ref. 20–29)**							
30–39	−0.370	1.466	−0.024	−0.252	0.801	−3.262	2.522
>40	−5.349	2.224	−0.284	−2.405	0.017	−9.735	−0.963
**Marital status (ref. single)**							
Married	7.395	1.325	0.485	5.581	<0.001	4.782	10.008
**Seniority (ref. intermediate)**							
Senior	2.236	1.788	0.109	1.250	0.213	−1.29	5.761
Primary	−0.716	1.441	−0.047	−0.497	0.620	−3.559	2.127
*R* ^2^	0.223						
Adjusted *R*^2^	0.203						

### The association between social support and depression and anxiety

Spearman correlation test showed that the total SSRS score, subjective social support score, objective social support score, and support utilization score among FHW were all negatively correlated with the SAS score and SDS score (*P* < 0.05) (Table 5). Multiple linear regression analysis suggested that a lower support utilization score was respectively significantly associated with high anxiety and depressive symptoms (β = −0.869, *P* = 0.024; β = −1.088, *P* = 0.035, respectively). Multiple linear regression analysis also showed that the total SSRS score, objective social support score, and support utilization score were associated with anxiety and depressive symptoms, which explained 8.9 and 14.9% of all variance, respectively (Tables 6, 7).

**TABLE 5:** Correlations between social support, anxiety, and depression.

Variables	SDS			SAS		
	*r*		*P*-value	*r*		*P*-value
Total SSRS score	−0.345		<0.001	−0.222		0.002
Subjective social support score	−0.260		<0.001	−0.156		0.027
Objective social support score	−0.257		<0.001	−0.176		0.013
Support utilization score	−0.335		<0.001	−0.268		<0.001

SAS, Self-rated Anxiety Scale; SDS, Self-rated Depression Scale; SSRS, Social Support Rate Scale.

**TABLE 6:** Regression analysis of the effects of social support on depression.

Variables	β (SE)	95% CI	*P*-value	Adjusted *R*^2^
Objective social support score	−0.066 (0.396)	−0.848, 0.715	0.867	0.149
Support utilization score	−1.088 (0.513)	−2.099, −0.076	0.035	
Total SSRS score	−0.353 (0.222)	−0.789, 0.084	0.113	

β, the coefficients; CI, confidence interval; SE, standard error; SSRS, Social Support Rate Scale.

**TABLE 7:** Regression analysis of the effects of social support on anxiety.

Variables	β (SE)	95% CI	*P*-value	Adjusted *R*^2^
Objective social support score	−0.039 (0.296)	−0.622, 0.544	0.896	0.089
Support utilization score	−0.869 (0.382)	−1.624, −0.115	0.024	
Total SSRS score	−0.136 (0.165)	−0.462, 0.190	0.412	

β, the coefficients; CI, confidence interval; SE, standard error; SSRS, Social Support Rate Scale.

## Discussion

To the best of our knowledge, this is the first study to investigate the relationship between the levels of social support and the prevalence of depression and anxiety among FHW during the COVID-19 pandemic in China. FHW are the direct providers of hospital services and the main force in controlling COVID-19. Understanding their level of social support and the relationship between psychological impact and social support can help Chinese hospital management and health policymakers take effective measures to further improve the mental health well-being of FHW, thus improving their professional performance and work efficiency.

### Prevalence of depression and anxiety

This study showed that during the COVID-19 pandemic, the prevalence of depressive and anxiety symptoms among FHW was 21.9 and 15.9%, respectively. However, using the same measurement as in our study, the prevalence of depressive and anxiety symptoms among FHW in the early stages of the COVID-19 pandemic was 35.8 and 22.4% (31), respectively, which was significantly higher than the population surveyed in our study. This is most likely associated with the deployment of psychological assistance services by the Chinese government. On 26 January 2020, the Ministry of Health of the Chinese Government issued a guideline for emergency psychological crisis intervention and counseling (32). On 2 February 2020, the state council of China set up a nationwide psychological assistance hotline to help people suffering from psychological disorders due to the epidemic (33). These programs are not only for patients with COVID-19 and the general public but also for all healthcare workers. Participants in this study received psychological assistance services before submitting questionnaires, which can reduce the prevalence of depression and anxiety.

### Factors affecting the level of depression and anxiety

Our findings showed that no significant difference was found in the effect of demographic factors (such as age, gender, seniority, and education level) on depression and anxiety symptoms. The main reasons can be related to the small sample size of this study and the relatively low proportion of FHW with depression and anxiety. Many FHW experiencing symptoms of anxiety and depression had mild degrees of depression and anxiety in our study. Among all the participants, only 12 (27.3%) had moderate and severe depression, and 3 (9.4%) had moderate and severe anxiety. A previous study reported that the anxiety levels in health workers did not vary significantly with age, education, and marital status (34). However, a recent meta-analysis revealed the prevalence of anxiety and depression was higher among females and nursing staff than among males and doctors during the COVID-19 pandemic (3). Nurses, FHW, female, young, and intermediate seniority were associated with a severe degree of depression and anxiety (6, 35). Furthermore, a significant causal relationship was found between depression and age and working on the frontline (36). Another study reported that working in an isolation ward or fever clinic was an independent risk factor for depression and anxiety among frontline pediatric nurses, whereas age and education level did not have any significant effect on depression and anxiety (37). The effect of demographic factors on depression and anxiety is controversial and more rigorously designed studies are required to further clarify this issue.

### Level of social support

Our study revealed that the total SSRS score of FHW was significantly lower than that of the general population. The three dimensions of social support (namely subjective social support, objective social support, and support utilization) of FHW were all significantly lower than the Chinese general population. In SSRS, subjective social support refers to the support received from family, friends, and colleagues. During the COVID-19 pandemic, the government-imposed quarantine policies and increased workloads limited the time that FHW could spend with family members and friends. Objective support refers to any type of visible or actual social support, especially economic assistance, received from any source, including the government, non-governmental organizations, religious groups, and local communities (38). The low social status of healthcare workers in China can limit their access to objective social support beyond family members (39). The lockdown policies imposed by the government can also limit their participation in community activities, which made it difficult for them to obtain community help. Support utilization refers to the degree of willingness to seek social support. Fear of stigma can make FHW reluctant to seek outside support (40). Moreover, excessive workloads and minimal vacations can also lead to extreme fatigue for FHW, which can limit their willingness to join in social interactions during breaks (41).

### Correlation between social support and depression and anxiety

We also found a negative correlation between the levels of social support and the severity of depressive and anxiety symptoms. The participants in our study who reported higher levels of social support were less likely to have symptoms of depression and anxiety, which indicated that social support is an important protective factor for the mental health of FHW. This was consistent with the results from previous studies (42, 43).

Subjective social support reflects the personal experience and feelings of social support (44). People with higher subjective social support score indicates that they receive adequate support, understanding, and respect from their family, friends, and colleagues. High subjective social support can help individuals to reduce loneliness and build a positive self-image, self-efficacy, and self-esteem, thus bringing more understanding, respect, courage, and professional achievements to themselves (45), which has a positive effect on reducing depression and anxiety of FHW.

Objective social support emphasizes the existence of visible social support (46). Those who scored higher on objective social support indicated that they received more visible help and support from the government, local communities, and non-governmental organizations. It also means they have extensive social networks. A high level of objective social support helps individuals to reduce the stress in work and life, and maintain good mental health, which can decrease the depressive and anxiety symptoms in FHW. Moreover, a wide social network can decrease the perceived threat of stressful events among FHW and reduce the physical reactions induced by stress, which also has a positive effect on reducing anxiety and depressive symptoms (47).

High social support utilization indicates a greater willingness to seek social support. This usually manifests as an emotional outpouring to family or friends or seeking help by participating in activities organized by the local community or religious groups. In our study, the higher the social support utilization of FHW, the less likely they were to have symptoms of depression and anxiety. This is consistent with the finding that a better connection with others can mitigate the harmful effects of stressful life events (48).

### Factors affecting the level of social support

In the present study, the level of social support for FHW positively correlated with age. One possible reason could be that healthcare workers over 30 years are more likely to have experienced severe acute respiratory syndrome (SARS) and middle east respiratory syndrome (MERS) outbreaks; therefore, they have more experience in seeking social support in the COVID-19 pandemic (49). Moreover, being older also means they have a wider social network and more access to social support than younger people (less than 30 years old). Individuals who had more social support generally had better mental health than those who had less (50).

Our findings also showed that being single was associated with a low level of social support among FHW. One possible reason is that married healthcare workers have higher quality and wider social networks than single healthcare workers because they can receive additional social support from their spouse and spouse’s family (51). These results are consistent with the study by Jaffar Abbas to some extent (52).

Interestingly, despite previous research showing differences in social support between male and female healthcare workers (17), our study showed that gender does not affect the level of social support. This can be because both male and female healthcare workers have longer working hours during the COVID-19 pandemic. Therefore, they did not have sufficient time to participate in family and social activities to seek social support (53). This may also explain why no difference was found in the social support between nurses and doctors in this study.

The results of this study showed that the department where FHW work and how long they worked on the frontline did not affect their social support because FHW feel so exhausted during the COVID-19 pandemic that they were reluctant to seek social support through social and family activities (54).

Previous studies have reported that healthcare workers with high education levels and seniority will receive more social support from patients and the social environment because of their high professional level and rich experience (55). However, the result of the present study showed that education level and seniority do not affect the social support of FHW. Further study is required to explain this phenomenon.

### Policy implications

Based on our findings, during COVID-19 pandemic, policy makers should: (1) reduce the working hours and workload of FHW and give them more time to participate in social and family activities; (2) pay more attention to the mental health of unmarried and young FHW and extend more help to alleviate the symptoms of depression and anxiety.

### Limitations

This study has several limitations. First, this study used self-report measures, hence there was a risk of information bias. Second, our study was a cross-sectional study that limited our ability to make statements on causality. In the absence of further follow-up studies, caution should be exercised regarding causality. Third, the income level and religious belief of FHW were not considered in this study, which has a certain relationship with social support. Further prospective and longitudinal studies with a large sample size are needed to assess the impact of social support levels on the mental health in the context of COVID-19 pandemic.

## Conclusion

In this study, we showed that 21.9 and 15.9% of FHW had depressive and anxiety symptoms, respectively. There was lower social support among FHW in comparison to the Chinese general population during the COVID-19 pandemic. The marital status and age had a major effect on social support. Social support was inversely associated with depression and anxiety. These findings signify that social support plays an important role in mental health, and health policymakers should pay more attention to the psychological status of FHW. Efforts should also be made to address their low level of social support, to reduce adverse psychological outcomes among FHW. More studies are required to determine how to improve the level of social support and mental health condition of FHW facing public health emergencies in the future.

## Data availability statement

The original contributions presented in this study are included in the article/supplementary material, further inquiries can be directed to the corresponding author.

## Ethics statement

The studies involving human participants were reviewed and approved by the Ethics Committee of Guangdong Provincial Hospital of Chinese Medicine (No. ZE2020-036). Written informed consent for participation was not required for this study in accordance with the national legislation and the institutional requirements.

## Author contributions

JZ and LL designed the study. JZ, LZ, and HC were responsible for the development and distribution of questionnaire. XY and XW were responsible for data auditing and data cleaning. JZ, CC, and LL analyzed and interpreted the data. CC and JZ revised the manuscript on the basis of comments from other authors. All authors have read and approved the final manuscript.

## Footnotes

1
https://www.wjx.cn
